# Solution processable and optically switchable 1D photonic structures

**DOI:** 10.1038/s41598-018-21824-w

**Published:** 2018-02-23

**Authors:** Giuseppe M. Paternò, Chiara Iseppon, Alessia D’Altri, Carlo Fasanotti, Giulia Merati, Mattia Randi, Andrea Desii, Eva A. A. Pogna, Daniele Viola, Giulio Cerullo, Francesco Scotognella, Ilka Kriegel

**Affiliations:** 1 Center for Nano Science and Technology@PoliMi, Istituto Italiano di Tecnologia, Via Giovanni Pascoli, 70/3, 20133 Milan, Italy; 2 Dipartimento di Chimica, Materiali e Ingegneria Chimica “Giulio Natta”, Politecnico di Milano, Piazza Leonardo da Vinci 32, 20133 Milano, Italy; 3 Dipartimento di Fisica, Politecnico di Milano, Piazza Leonardo da Vinci 32, 20133 Milano, Italy; 4 Department of Nanochemistry, Istituto Italiano di Tecnologia (IIT), via Morego, 30, 16163 Genova Genova, Italy; 5 The Molecular Foundry, Lawrence Berkeley National Laboratory, Berkeley, California, 94720 United States

## Abstract

We report the first demonstration of a solution processable, optically switchable 1D photonic crystal which incorporates phototunable doped metal oxide nanocrystals. The resulting device structure shows a dual optical response with the photonic bandgap covering the visible spectral range and the plasmon resonance of the doped metal oxide the near infrared. By means of a facile photodoping process, we tuned the plasmonic response and switched effectively the optical properties of the photonic crystal, translating the effect from the near infrared to the visible. The ultrafast bandgap pumping induces a signal change in the region of the photonic stopband, with recovery times of several picoseconds, providing a step toward the ultrafast optical switching. Optical modeling uncovers the importance of a complete modeling of the variations of the dielectric function of the photodoped material, including the high frequency region of the Drude response which is responsible for the strong switching in the visible after photodoping. Our device configuration offers unprecedented tunability due to flexibility in device design, covering a wavelength range from the visible to the near infrared. Our findings indicate a new protocol to modify the optical response of photonic devices by optical triggers only.

## Introduction

Solution processed photonic crystals offer the option of easy device fabrication on various substrates, with the additional benefit of introducing materials with diverse and complementary features. For example, the integration of plasmonic nanoparticles into a photonic crystal gives a new degree of freedom in the design of its optical response^1–3^. Recently, heavily doped semiconductor nanoparticles that show plasmonic response in the near infrared (NIR) have attracted the interest of the scientific community. Such materials are very sensitive to changes in dielectric function induced by variations in their carrier density through doping control^4–8^. This, in turn, directly modifies their Drude response and ultimately results in a blue shift of their near infrared plasmon resonance. Notably, in recent years there have been proposals to add reversibility to this effect by introducing and extracting extra carriers via capacitive charging, a process that permits to switch the optical response of the material in the NIR by modulating the amount of charges injected/extracted via an applied bias^9–14^. Furthermore, one can easily introduce extra carriers in such materials via optical triggers, i.e. photodoping^9,10,15–18^. In such process, photons with energies larger than the optical band gap promote electrons to the conduction band, leaving holes in the valence band. In the presence of a chemical hole scavenger the latter are extracted, leaving the system behind with additional electrons in their conduction band.

The integration of heavily doped semiconductor nanocrystals in one dimensional (1D) photonic crystals has been shown previously, for example by Puzzo *et al*. who reported silicon dioxide (SiO_2_)/antimonium tin oxide (ATO) and ATO/titanium dioxide (TiO_2_) 1D photonic crystals^19,20^. However, the exploitation of their tunable plasmonic response has not been demonstrated yet. This is particularly interesting as the carrier density modulation results in the active manipulation of their frequency dependent dielectric constant which in turn modulates the refractive index that determines the photonic bandgap of the device. Thus, the modulation of the NIR plasmonic response of the nanoparticle film can be employed to modulate the overall optical response of the photonic system, with a two-fold effect: a change in the NIR plasmonic response^21^, as well as a variation of the photonic band gap^22–25^.

In this work we study, for the first time, such plasmonic/photonic effect in a photonic crystal composed of alternating nanoparticle layers of SiO_2_ and indium tin oxide (ITO). The latter is a very prominent heavily doped semiconductor with carrier densities in the range of 10^21^ cm^−3^ in which the reversible tunability of its NIR plasmonic response by various means has been demonstrated^16,26–28^. The photonic device covers the entire spectrum from the visible (photonic band gap) to the NIR (free carrier response in ITO), supported by optical modeling. We demonstrate the switching of its optical response via photodoping in steady state and on ultrafast time scales, together taking a step towards a solution processed, contactless, all-optical switching device.

## Results and Discussion

**Figure 1:**
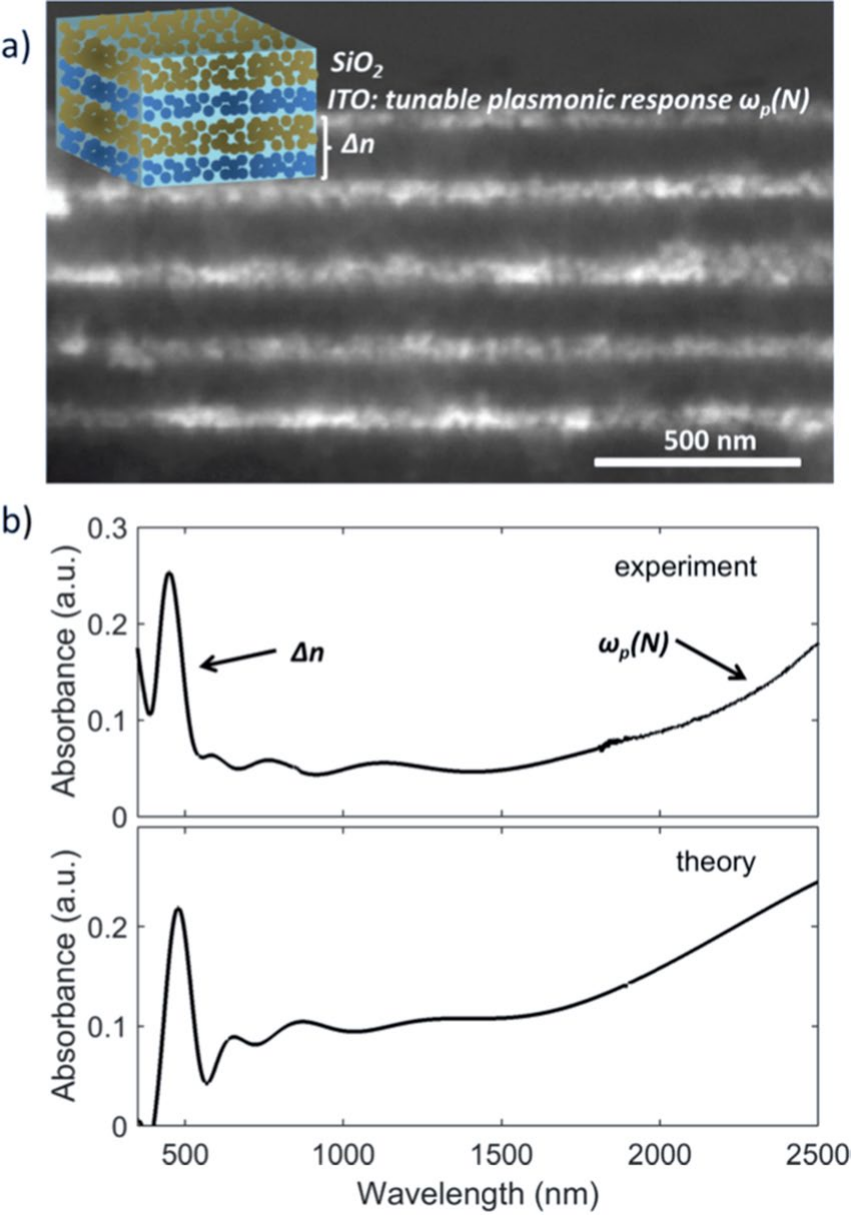
(**a**) SEM cross section of the SiO_2_/ITO 1D photonic crystal. The bright layers correspond to the higher contrast ITO nanoparticle layer, the darker layers to SiO_2_. Inset: sketch of the SiO_2_/ITO 1D photonic crystal indicating the contribution to the optical response: *Δn* corresponds to the refractive index contrast, which is responsible for the photonic bandgap in the visible, while the high absorption in the near infrared is due to free carriers (i.e. plasmonic response) of the ITO nanoparticles depicted by the plasma frequency as a function of the carrier density *N* (*ω*_*p*_*(N)*). (**b**) Experimental (upper panel) and theoretical (lower panel) absorption spectrum of the 1D photonic structure at normal incidence.

Photonic crystals have been fabricated by solution processing of alternating layers from dispersion of SiO_2_ and ITO nanoparticles, respectively. In Fig. 1a we report the SEM cross section of the SiO_2_/ITO 1D photonic crystal made of 5 bilayers. The relatively darker regions correspond to the SiO_2_ layers, while the brighter ones to the ITO layers due to the lower atomic weight of silicon with respect to indium and tin. The average layer thickness for SiO_2_ is 120 nm, while the one for ITO is 60 nm. In the upper panel of Fig. 1b we show the measured absorption spectrum, at normal incidence, of the fabricated SiO_2_/ITO photonic crystal, displaying a peak at around 500 nm and intense absorption in the near infrared. The peak in the visible can be ascribed to the photonic band gap of the photonic crystal as a result of the refractive index contrast *Δn* between the alternating nanoparticle layers. We report in the Supplementary Material the transmission of the photonic crystal as a function of the incidence angle of light (Figure S1), showing the blue shift of the photonic band gap by increasing the angle, in agreement with the Bragg-Snell law^1^. The tail from about 1500 nm towards longer wavelengths instead arises from the plasmonic response of the ITO nanoparticles, brought about by the free carrier density *N* in the range of 10^20^–10^21^ cm^−3^^16,26–28^. We remark here that for the sake of simplicity we give the optical response of the system in absorbance, rather than transmittance, although the visible peak of the photonic bandgap is not actually due to absorption. Absorbance however, takes into account all processes occurring in the sample due to absorption and reflection.

We modeled the optical response of the photonic crystal by implementing two different models that account for the respective spectral contributions: firstly, we implemented the transfer matrix theory, which takes into account the alternating refractive index of the periodic structure, and secondly we integrated the Drude model and the Maxwell-Garnet effective medium approximation (MG-EMA) to consider the contribution of the ITO nanoparticle films to the dielectric response of the photonic crystal^29–31^. The MG-EMA theory is implemented to account for a dense distribution of particles dispersed in a dielectric matrix, considering that the layer of ITO actually consists of ITO nanoparticles and air voids. The effective dielectric function is then given as:

1 $${\varepsilon }_{eff,ITO}(\omega )={\varepsilon }_{Air}\frac{2(1-{\delta }_{ITO}){\varepsilon }_{Air}-(1+2{\delta }_{ITO}){\varepsilon }_{ITO}(\omega )}{2(2+{\delta }_{ITO}){\varepsilon }_{Air}+(1+{\delta }_{ITO}){\varepsilon }_{ITO}(\omega )},$$

where δ_ITO_ is the so-called fill factor, representing the fraction of the volume of the inclusions occupying the volume of the film, while *ε*_*ITO*_*(ω)* is the frequency dependent Drude dielectric function of ITO and *ε*_*Air*_ = (1.00059)^2^. Consequently, we obtain the effective refractive index by

2 $${n}_{eff,ITO}^{2}(\omega )={\varepsilon }_{eff,ITO}(\omega ).$$

The dielectric function of ITO is described by the Drude theory, which takes into account the contribution from the free carriers to the optical response and is given by:

3 $${\varepsilon }_{ITO}(\omega )={\varepsilon }_{1}(\omega )+i{\varepsilon }_{2}(\omega ),$$

where the real part is given as

4 $${\varepsilon }_{1}(\omega )={\varepsilon }_{\infty }-\frac{{\omega }_{P}^{2}}{({\omega }^{2}-{{\rm{\Gamma }}}^{2})},$$

and the imaginary part as

5 $${\varepsilon }_{2}(\omega )=\frac{{\omega }_{P}^{2}{\rm{\Gamma }}}{\omega ({\omega }^{2}-{{\rm{\Gamma }}}^{2})},$$

where ω is the frequency and Γ is the damping (inverse of the carrier relaxation time). The plasma frequency ${\omega }_{P}$ is given as

6 $${\omega }_{P}=\sqrt{\frac{N{e}^{2}}{{m}^{\ast }{\varepsilon }_{0}}},$$

where *N* is the carrier density, *e* is the electron charge, m* is the carrier effective mass and ε_0_ is the vacuum permittivity. This relation shows clearly that the plasmonic response is proportional to the square root of the carrier density, resulting in a doping-dependent plasma frequency *ω*_*p*_*(N)*.

We also implemented the effective refractive index of the SiO_2_ nanoparticle layer for the calculation of the alternating structure with the transfer matrix method. For details on the transfer matrix method please refer to the methods section^32–34^. The lower panel of Fig. 1b reports the calculated absorption spectrum of the 1D photonic crystal, showing remarkable overlap with the experiment. Note that we used a film thickness of 110 nm for the SiO_2_ layer to match the position of the photonic bandgap. We obtained the best fit by employing a fill factor for the ITO nanoparticle film of δ_ITO_ = 0.3. We used a significantly higher fill factor (0.95) for the SiO_2_ films due to the much higher density, as observed in the SEM image in Fig. 1a and in the Supplementary Materials Figure S3. We extracted a plasma frequency of *ω*_*P*_ = 9000 cm^−1^ and from the latter determined the carrier density of 3.7∙10^20^ cm^−3^, which is in agreement with common ITO samples^16,26–28^.

**Figure 2:**
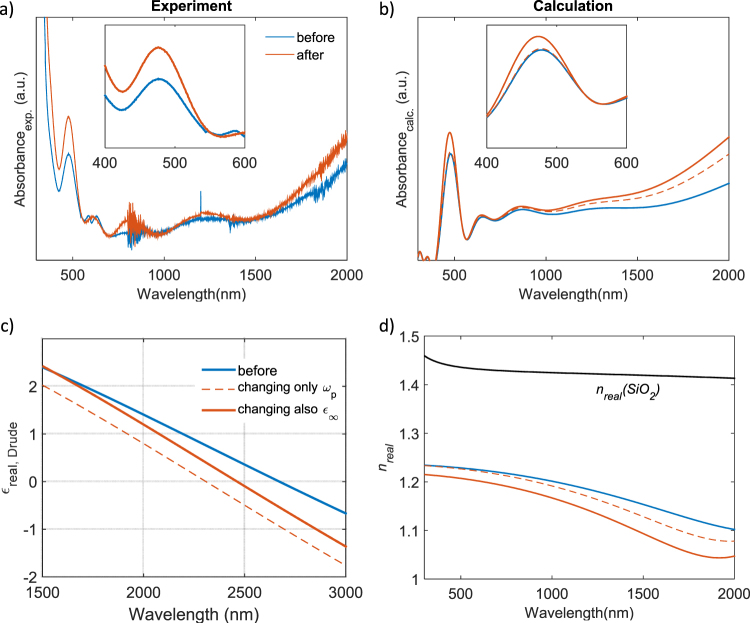
(**a**) Experimental absorption spectrum of the SiO_2_/ITO photonic crystal, before and after UV exposure (photodoping); (**b**) calculation of the absorption spectrum with the transfer matrix method, integrated with the Drude model and the Maxwell-Garnet effective medium approximation; (**c**) real part of the Drude dielectric function of the ITO nanoparticles; (**d**) real part of the refractive index of the ITO nanocomposite film as employed in the calculation. Black curve: SiO_2_, blue to red curves: ITO nanocomposite.

Having assigned the spectral signatures of our device structure, we performed a photodoping experiment by exposing the ITO/SiO_2_ photonic structure to UV light (310 nm LED) for five minutes and measuring its absorbance before and after illumination (Fig. 2). We observe an increase of the absorption in the NIR spectral range after illumination due to the enhanced carrier density related to *ω*_*p*_*(N)* (red curve in Fig. 2a) Notably, we also observe a significant effect on the region of the photonic bandgap (see inset to Fig. 2a), related to the difference in the refractive indexes of the two alternating layers. To obtain a better understanding of these spectral changes we modelled this system also after photodoping, and the results of this simulation are given in Fig. 2b–d. The blue curve gives the same modelling result of the photonic device as shown in Fig. 1b (lower panel). Figure 2c shows the Drude dielectric function of the ITO nanoparticles, showing the cross-over from positive to negative values in the so called epsilon near zero regime^35,36^. After UV light exposure, in our model we artificially increase the plasma frequency *ω*_*p*_*(N)*, mimicking the increased carrier density after photodoping. The simulation (Fig. 2b, dashed curve) shows that we were able to reproduce the variation observed in the NIR by adjusting this parameter, indicating an important role of the enhanced carrier density on the optical spectra. However, this simple picture does not permit to reproduce the shift observed in the region of the photonic bandgap. When looking at the real part of the refractive index *n*_*real*_ of our nanoparticle film, as given in Fig. 2d, it becomes clear that the refractive index contrast *Δn* is barely altered in the visible spectral range (red dashed curves versus blue curves, respectively) when increasing *ω*_*p*_*(N)*. Interestingly, after inducing a refractive index change *Δn* after varying further the high frequency dielectric constant (*ε*_∞_), we are able to explain the experimental changes of the photonic bandgap in the visible spectral range (Fig. 2b), as this largely alters the refractive index contrast with SiO_2_ (black curve in Fig. 2d). Actually, it has been shown that increased doping influences also the region of the interband transitions in relation to the Moss-Burstein shift of the bandgap or, in other words, it increases Pauli blocking due to the occupation of additional levels in the conduction band^37^. The sample becomes more transparent in the high frequency region resulting in an increase in the high frequency dielectric constant (*ε*_∞_). Our results highlight the paramount importance of a careful study of the variation of the dielectric function upon photodoping of such materials. In particular, although a major role has been attributed to the increase in charge carrier density that dictates the optical modulation of charged metal oxide structures^38^, our results prove unambiguously that also the high frequency dielectric constant is markedly altered by the photodoping process. Indeed, when looking at the Drude response of the varied dielectric functions after photodoping, one can see a strong influence in particular on the epsilon near zero regime of the real part of the Drude dielectric function (Fig. 2c), highlighting the importance to study in detail the variations of the dielectric response to understand the effects of carrier injection upon photodoping. Moreover, our results illustrate how all-optical control over the epsilon near zero regime can be employed to design photo-switchable devices. Note that the effective refractive index of the photonic crystal decrease slightly upon photodoping (Fig. S2).

**Figure 3:**
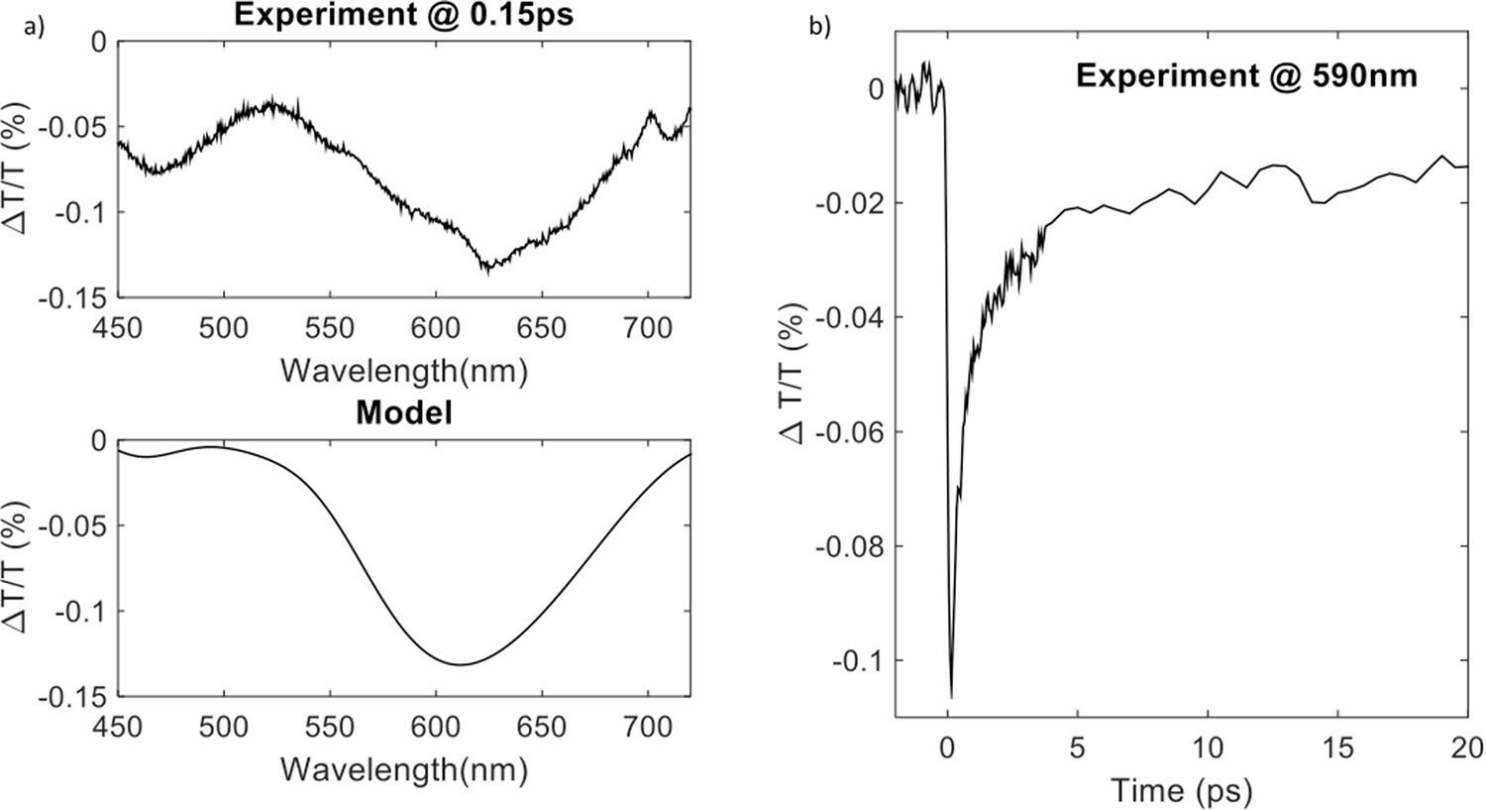
(**a**) Differential transmission spectrum at 0.15 ps pump-probe delay after ultrafast photoexcitation of the 1D photonic crystal at 260 nm (4.77 eV). Upper panel displays the model and lower panel displays the experiment. (**b**) Differential transmission dynamics at 590 nm.

It has been shown in previous works that the photodoping can also be performed on ultrafast time scales, initiating changes in the optical properties with picosecond dynamics. For instance, in fluorine and indium doped cadmium oxide (FICO) nanocrystals an ultrafast modulation of the plasmon resonance has been shown after band edge excitation due to the ultrafast photodoping^39^. The authors measured a monoexponential recovery time within several picoseconds, indicative of the recombination of photogenerated electrons and holes. These measurements thus detect the temporarily increased carrier density after photodoping as long as the electron is placed in the conduction band and before recombining with the photogenerated hole. Similar results have been observed also in indium cadmium oxide nanocrystals^35^ and in ITO nanopillars^26^. We performed ultrafast photodoping on our photonic crystal device by pumping the sample with a 150 fs laser pulse at 266 nm (4.66 eV), well above the band gap of ITO (about 4 eV). Figure 3 shows the ΔT/T spectrum of the SiO_2_ – ITO 1D photonic crystal at around 150 fs pump-probe delay and the ΔT/T dynamics at 590 nm close to the maximum of the photonic bandgap. Note that the specifications of the photonic structure were not exactly the same as in the previous example and therefore the photonic bandgap shifted to higher wavelengths. Remarkably, we observe an ultrafast recovery time of the signal within about 20 picoseconds, and a change of the transmission of approximately 10% at moderate pump intensities (0.5 mJ/cm^2^). In addition, we show here that we can simulate the differential transmission spectrum by employing the extracted parameters from the steady state model, as used above and implementing the transmission changes from the transfer matrix method calculated as:

7 $$\frac{{\rm{\Delta }}T}{T}=\frac{{T}_{ON}-{T}_{OFF}}{{T}_{OFF}}$$

where *T*_*ON*_ accounts for the transmission of the white light through the sample after photo-excitation at 260 nm. We obtained a good agreement between the experimental and simulated spectra. Similarly to the reports on the ultrafast photodoping of FICO nanocrystals, in our case a change of the high frequency dielectric constant has been used to describe the ultrafast response. Although for FICO nanocrystals a positive variation of approximately 0.4 was observed, we see here a negative variation of the same magnitude. The difference in sign between the two experiments might be attributed to the different system environment:a dilute solution in the case of FICO, and, densely packed films in the case of ITO. Additionally, differences in the material band structure might also play a role. To clarify this point further studies of the ultrafast optical response of ITO nanocrystals in the near infrared are required. Nevertheless, our results show that ultrafast photodoping of ITO nanocrystals translates to a transient change of the photonic bandgap in the visible spectral range.

## Conclusion

In this paper we report the solution processed fabrication of photo-switchable 1D photonic crystals with response in a dual range of wavelengths: the photonic stop band in the visible range and the plasmonic resonance in the near infrared. The incorporation of heavily doped semiconductor nanoparticle layers adds an additional degree of tunability to the device due to charge injection triggered by photodoping of the ITO nanoparticles. The all-optical switching is obtained by exposing the device to UV light which results in a modification of both the photonic bandgap and the plasmon resonance. Optical modeling revealed that the photodoping has an effect not only on the plasma frequency *ω*_*p*_*(N)* that depends on the carrier density, but also on the high frequency refractive index, most probably due to the Moss-Burstein effect of heavily doped semiconductors. This highlights that advanced optical modeling is required to extract fundamental variations in the dielectric function of photo-switchable nanoparticles. Ultrafast switching times of the photonic bandgap in the visible have been extracted after transient photodoping, suggesting our system for ultrafast optical signal processing with high transmission changes and recovery time in the picosecond time frame. Taken together, our results suggest a new optically switchable system, in which the optical response can be varied in the stationary and transient regimes in a contactless manner.

## Methods

### Nanoparticles

SiO_2_ nanoparticles were purchased from Sigma-Aldrich (Ludox SM-30) and were diluted with distilled water to a final concentration of 5 wt. %. The average size of the nanoparticles is 8 nm. ITO nanoparticles were purchased from Gentech Nanomaterials and were diluted with distilled water to a final concentration of 5 wt. %. The average size of the nanoparticles is 20–30 nm. The dispersions were sonicated for 60 minutes at room temperature and filtered with a 0.45 µm PVDF filter. We show in the Supplementary Material (Figure S1).the SEM images of the SiO_2_ and ITO films, deposited following the same procedure used for the fabrication of the photonic crystals (see below).

### Fabrication of the 1D photonic crystal

A glass substrate was washed in isopropanol and then in acetone in a sonicating bath for 5 minutes. Then, organic contaminants were removed from the substrate via an oxygen plasma treatment.

The photonic crystal was fabricated using a spin coater model Laurell WS-400- 6NPP-Lite. The rotation speed for the deposition was 2000 rotations per minute. After each deposition, the sample has been annealed for 10 minutes at 350 °C on a hot plate under the fume hood.

### Scanning electron microscope characterization

The microscope was a Tescan MIRA3. The measurement were performed at a voltage of 5 kV and backscattered electrons was detected. The sample was covered with carbon paste to improve conductivity.

### Spectroscopic measurements

The transmission spectrum of the photonic crystal has been measured with a Perkin Elmer spectrophotometer Lambda 1050 WB. The ultrafast spectroscopy measurement has been performed with a laser system based on an amplified Ti:Sapphire laser (Coherent Libra), with a maximum output energy of about 800 µJ, 1 kHz repetition rate, and 800-nm central wavelength. The pulse duration is about 100 fs. Excitation pulses at 266 nm were obtained via the sum frequency of the fundamental frequency pulse and its second harmonic in a β-Barium borate (BBO) crystal. Pump pulses were focused to a 200 µm diameter spot. Probing was achieved in the visible by using white light generated in a thin sapphire plate. Chirp-free differential transmission (ΔT/T) spectra were collected by using an optical multichannel analyser with a dechirping algorithm.

### CW Photodoping experiment

After UV irradiation of the sample with a LED at 310 nm for 5 minutes, an absorption spectrum has been acquired. The UV irradiation and the absorption measurement have been performed at room temperature in air.

### Transfer Matrix Method

The transmission spectra of the photonic crystal were calculated by employing the transfer matrix method, widely used for modeling the optical response of multilayers^32–34,40–42^. The amplitude of the electric and magnetic fields are calculated after the light wave propagates through the multilayer stacks considering the Maxwell equations with the proper boundary conditions. The matrix product of the characteristic transmission matrices through each layer gives the overall transmission, as it calculates the electric and magnetic fields at the output of the photonic structure. The assumption of isotropic, non-magnetic systems is valid for most dielectric materials and a normal incidence angle was implemented. The following system of output amplitudes has been solved:

8 $$[\begin{array}{c}{E}_{0}\\ {H}_{0}\end{array}]={M}_{1}\cdot {M}_{2}\cdot \ldots \cdot {M}_{m}[\begin{array}{c}{E}_{m}\\ {H}_{m}\end{array}]=[\begin{array}{c}{m}_{11}\\ {m}_{21}\end{array}\begin{array}{c}{m}_{12}\\ {m}_{22}\end{array}]\,[\begin{array}{c}{E}_{m}\\ {H}_{m}\end{array}]$$

Here, *E*_0_ and *H*_0_ depict the amplitudes of electric and magnetic fields at the output, while *E*_*m*_ and *H*_*m*_ are the amplitudes at the input. The matrices for each layer *j* are then given by:

9 $${M}_{j}=[\begin{array}{cc}{A}_{j} & {B}_{j}\\ {C}_{j} & {D}_{j}\end{array}],$$

with *j* = (1, 2, …, *m*) and the elements of the transmission matrix *ABCD*:

10 $${A}_{j}={D}_{j}=\,\cos ({\varphi }_{j}),\,{B}_{j}=\,-(\frac{i}{{p}_{j}})\sin ({\varphi }_{j}),\,{C}_{j}=-i{p}_{j}\,{\sin }({\varphi }_{j}).$$

For normal incidence the phase variation in the *j*-fold layer simplifies to ${\varphi }_{j}\,=\,(\frac{2\pi }{\lambda }){n}_{j}{d}_{j}$. *n*_*j*_ an*d d*_*j*_ are determined through the phase variation *ϕ*_*j*_, and represent the effective refractive index and the thickness of the layer *j*, respectively. The coefficients are ${p}_{j}=\sqrt{\frac{{\varepsilon }_{j}}{{\mu }_{j}}}$ for the transverse electric wave.

The transmission *t* is then determined by:

11 $$t=\frac{2{p}_{s}}{({m}_{11}+{m}_{12}{p}_{0}){p}_{s}+({m}_{11}+{m}_{12}{p}_{0})}$$

where *p*_*s*_ is for the substrate and *p*_0_ is for air. And the final light transmission given as:

12 $$T=\frac{{p}_{0}}{{p}_{s}}{|t|}^{2}$$

where *p*_*s*_ refers to the substrate and *p*_0_ to air.

## Electronic supplementary material

Supplementary Material
