# Supplementary material for: Solution processable and optically switchable 1D photonic structures
Source: Sci Rep. 2018 Feb 23;8:3517. doi: 10.1038/s41598-018-21824-w (PMC5824833; doi:10.1038/s41598-018-21824-w)
Supplement: Supplementary file 1 — Supplementary Material [file 41598_2018_21824_MOESM1_ESM.docx]

Supplementary material for

**Solution processable and optically switchable 1D photonic structures**

Giuseppe M. Paternò, Chiara Iseppon, Alessia D’Altri, Carlo Fasanotti, Giulia Merati, Mattia Randi, Andrea Desii, Eva A. A. Pogna, Daniele Viola, Giulio Cerullo, Francesco Scotognella, Ilka Kriegel

*Angular dependent transmission of the SiO_2_/ITO photonic crystal*. We have measured the light transmission of the SiO_2_/ITO photonic crystal as a function of the angle of the incident light (0 degrees corresponds to normal incidence). In Figure S1 we show the blue shift of the photonic band gap as a function of the angle, in agreement with the Bragg-Snell law ^2^. The position of the photonic band gap is not exactly the same of the one in Figure 1, since we have used a different setup and we have impinged the sample with a slightly different spot size [The setup used here is a tungsten lamp and a Stellarnet concave grating spectrometer with a spectral resolution of 1.5 nm].

Figure S1. Light transmission of the SiO_2_/ITO photonic crystals as a function of the angle of incidence of light.

*Effective refractive index of the photonic crystal*. We have taken the refractive index dispersions of the two layers (i.e. SiO_2_ and ITO) and put them in the Lorentz-Lorenz equation. Such equation for *n* materials can be written as

$$\frac{n^{2}-1}{n^{2}+2}\left( \frac{m}{\rho} \right)=\frac{n_{1}^{2}-1}{n_{1}^{2}+2}\left( \frac{m_{1}}{\rho_{1}} \right)+\frac{n_{2}^{2}-1}{n_{2}^{2}+2}\left( \frac{m_{2}}{\rho_{2}} \right)+..$$

with *m* mass and $\rho$ specific weight of mixture components ^1^. In our case, we can write the equation in terms of filling factor of SiO_2_ (0.647) and of ITO (0.353) to have

$$\frac{n^{2}-1}{n^{2}+2}=\frac{n_{{SiO}_{2}}^{2}-1}{n_{{SiO}_{2}}^{2}+2}0.647+\frac{n_{ITO}^{2}-1}{n_{ITO}^{2}+2}0.353$$

We report in Figure S2 the real part of the refractive indexes of the materials, where ITO pd means photodoped ITO. *n_eff_* is the effective refractive index of the photonic crystal, while *n_eff,2_* is the effective refractive index of the photodoped photonic crystal. We observed taht, due the change of the real part fo the ITO refractive index, the photonic crystal effective refractive index shows a slight decrease that explained the mild blue shift of the gap and, because of the increased value of $\Delta n=n_{{SiO}_{2}}-n_{ITO}$ , an increase of the photonic band gap intensity observed in the experimental data in Figure 2a.

Figure S2. Real part of the refractive index of the SiO_2_ layer, ITO layer and photodoped ITO (ITO pd). Real part of the effective refractive of the SiO_2_/ITO photonic crystal (*n_eff_*) and of the photodoped photonic crystal (*n_eff,2_*)

*Scanning electron microscope (SEM) images of SiO_2_ and ITO nanoparticle films*. The films have been deposited via spin coating, at a speed of 2000 rotations per minute, with 5% dispersions of nanoparticles in water.

a)
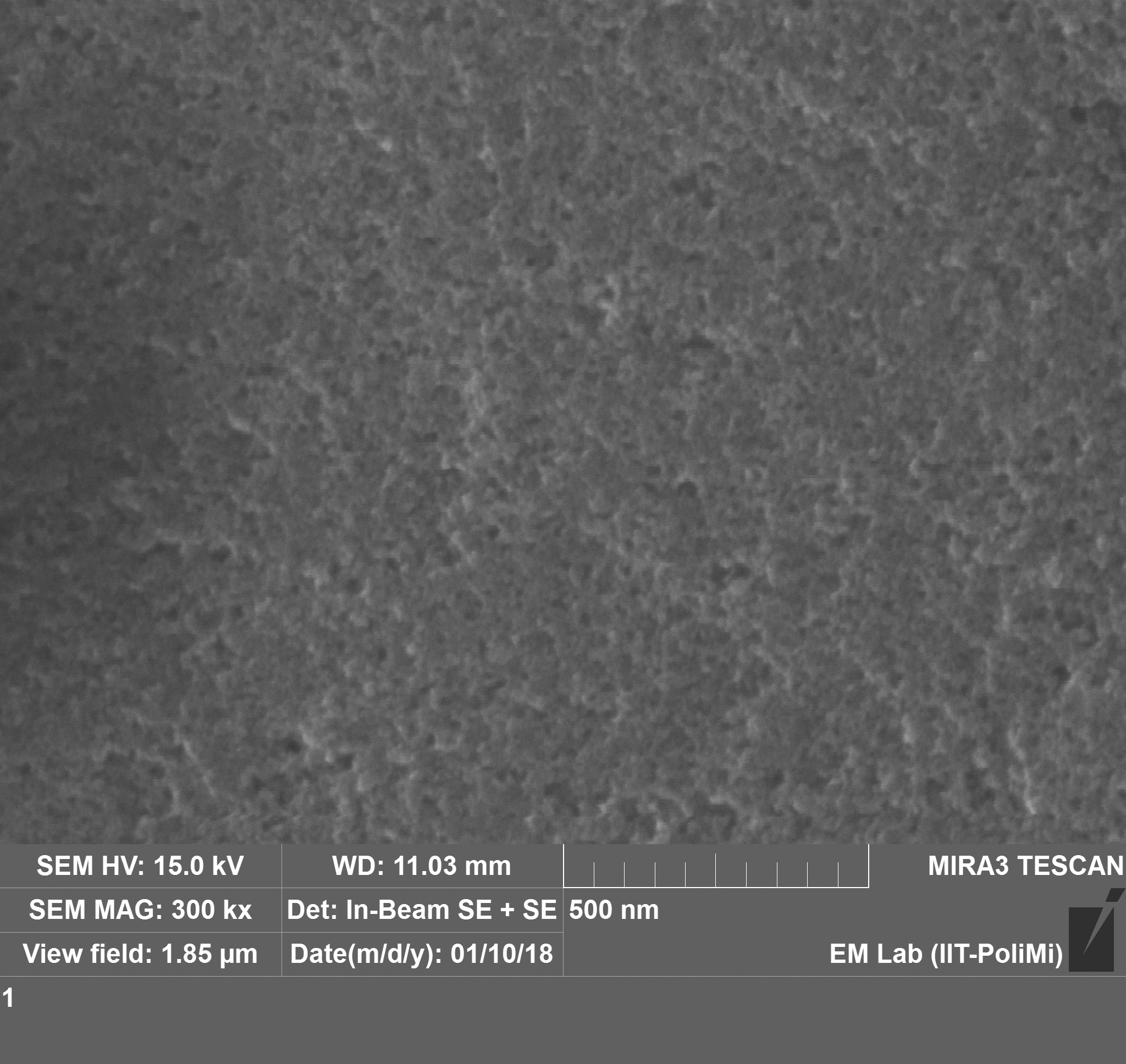


b)
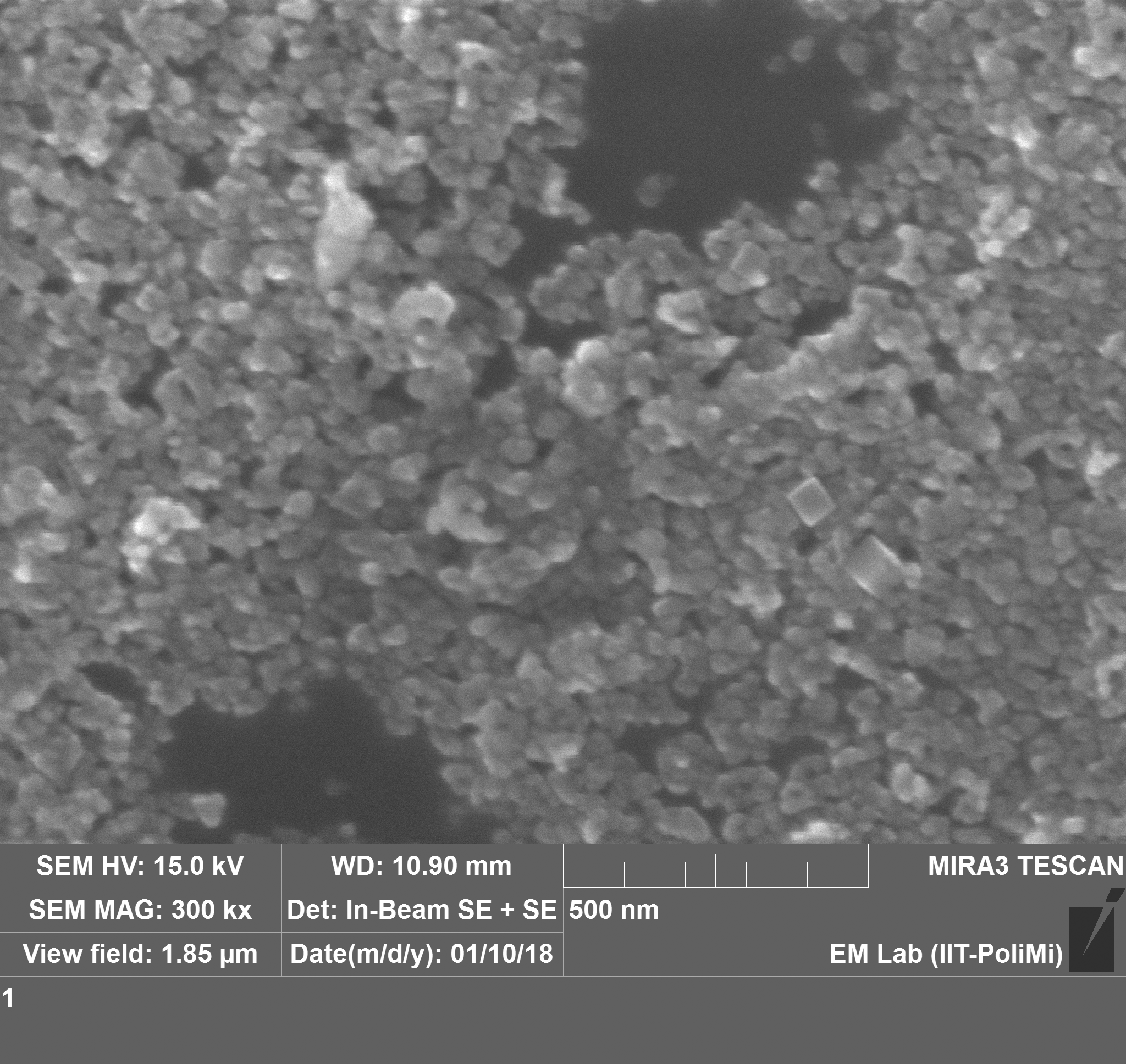


Figure S1. SEM images of (a) SiO_2_ nanoparticle and (b) ITO nanoparticle films

The ITO  image is better resolved because of the higher atomic number of indium.

**References**

1. Tikhonov, E. A., Lyamets, A. K. & Malyukin, Y. V. Refractive index of nanoscale thickness films measured by Brewster refractometry. *ArXiv150404262 Phys.* (2015).

2. Morandi, V., Marabelli, F., Amendola, V., Meneghetti, M. & Comoretto, D. Colloidal Photonic Crystals Doped with Gold Nanoparticles: Spectroscopy and Optical Switching Properties. *Adv. Funct. Mater.* **17,** 2779–2786 (2007).
